# Augmented mitochondrial energy metabolism is an early response to chronic glucose stress in human pancreatic beta cells

**DOI:** 10.1007/s00125-020-05275-5

**Published:** 2020-09-22

**Authors:** Isabelle Chareyron, Stefan Christen, Sofia Moco, Armand Valsesia, Steve Lassueur, Loïc Dayon, Claes B. Wollheim, Jaime Santo Domingo, Andreas Wiederkehr

**Affiliations:** 1 Nestlé Institute of Health Sciences, Nestlé Research, EPFL Innovation Park, Lausanne, Switzerland; 2 Ecole Polytechnique Fédérale de Lausanne, Lausanne, Switzerland; 3 Department of Cell Physiology and Metabolism, University Medical Center, Geneva, Switzerland

**Keywords:** Beta cells, Calcium, Human islets, Metabolomics, Mitochondria

## Abstract

**Aims/hypothesis:**

In islets from individuals with type 2 diabetes and in islets exposed to chronic elevated glucose, mitochondrial energy metabolism is impaired. Here, we studied early metabolic changes and mitochondrial adaptations in human beta cells during chronic glucose stress.

**Methods:**

Respiration and cytosolic ATP changes were measured in human islet cell clusters after culture for 4 days in 11.1 mmol/l glucose. Metabolomics was applied to analyse intracellular metabolite changes as a result of glucose stress conditions. Alterations in beta cell function were followed using insulin secretion assays or cytosolic calcium signalling after expression of the calcium probe YC3.6 specifically in beta cells of islet clusters.

**Results:**

At early stages of glucose stress, mitochondrial energy metabolism was augmented in contrast to the previously described mitochondrial dysfunction in beta cells from islets of diabetic donors. Following chronic glucose stress, mitochondrial respiration increased (by 52.4%, *p* < 0.001) and, as a consequence, the cytosolic ATP/ADP ratio in resting human pancreatic islet cells was elevated (by 27.8%, *p* < 0.05). Because of mitochondrial overactivation in the resting state, nutrient-induced beta cell activation was reduced. In addition, chronic glucose stress caused metabolic adaptations that resulted in the accumulation of intermediates of the glycolytic pathway, the pentose phosphate pathway and the TCA cycle; the most strongly augmented metabolite was glycerol 3-phosphate. The changes in metabolites observed are likely to be due to the inability of mitochondria to cope with continuous nutrient oversupply. To protect beta cells from chronic glucose stress, we inhibited mitochondrial pyruvate transport. Metabolite concentrations were partially normalised and the mitochondrial respiratory response to nutrients was markedly improved. Furthermore, stimulus–secretion coupling as assessed by cytosolic calcium signalling, was restored.

**Conclusion/interpretation:**

We propose that metabolic changes and associated mitochondrial overactivation are early adaptations to glucose stress, and may reflect what happens as a result of poor blood glucose control. Inhibition of mitochondrial pyruvate transport reduces mitochondrial nutrient overload and allows beta cells to recover from chronic glucose stress.

**Figure:**
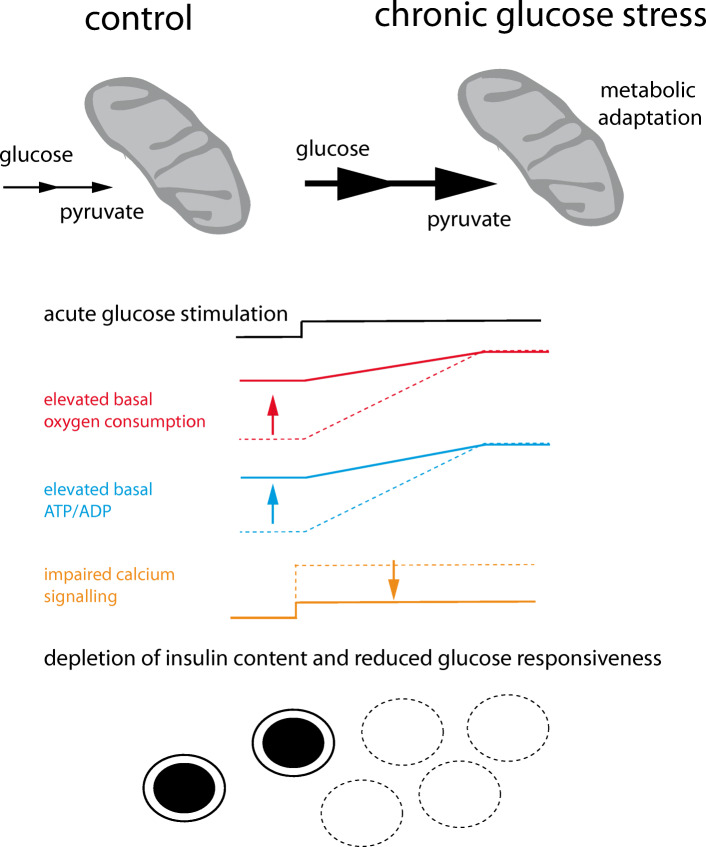
Graphical abstract

**Electronic supplementary material:**

The online version of this article (10.1007/s00125-020-05275-5) contains supplementary material, which is available to authorized users.

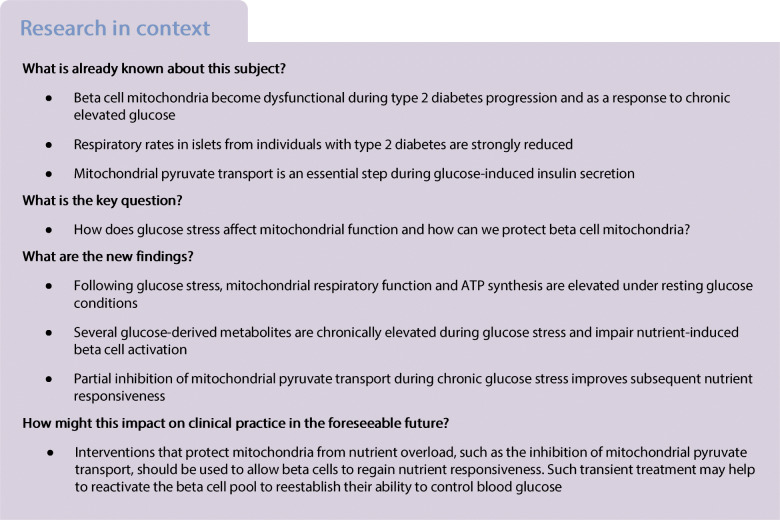



## Introduction

Following a meal, the pancreatic beta cell releases insulin, which lowers blood glucose. Beta cell failure or the inability of beta cells to compensate for insulin resistance leads to impaired fasting glucose and/or impaired glucose tolerance [1]. Over the time course of years, beta cell function and/or mass progressively declines, predominantly as a result of de-differentiation and long-term apoptosis, with further deterioration of glucose homeostasis eventually resulting in type 2 diabetes [2, 3].

Beta cells sense glucose through nutrient uptake and metabolism [4, 5]. Mitochondria play an essential role in glucose sensing, as they link glucose metabolism to the formation of ATP and other coupling factors that induce and regulate insulin secretion [6, 7]. Pyruvate generated by glycolysis enters mitochondria through the mitochondrial pyruvate transporter [5, 8–11] an essential step in beta cell activation [11–13].

Glucose stimulation accelerates oxidative metabolism and respiration, causing enhanced mitochondrial ATP synthesis [5, 8, 14, 15]. Stimulation of mitochondrial energy metabolism increases the cytosolic ATP/ADP ratio [14, 16, 17]. This promotes closure of K_ATP_ channels [18] and depolarisation of the plasma membrane, which leads to Ca^2+^ influx triggering insulin granule exocytosis [19]. In parallel, pyruvate stimulates anaplerosis [5, 9, 17] and thereby rapidly elevates the levels of tricarboxylic acid (TCA) cycle intermediates and other metabolites linked to glucose metabolism [9, 20–22]. Metabolite cycling between mitochondria and the cytosol generates cytosolic signals that amplify insulin secretion [23–25].

Chronic exposure of beta cells to elevated glucose concentrations impairs insulin secretion both in vivo and in vitro [2, 26–29]. Mitochondrial defects are known to prevent coupling between glucose metabolism and insulin secretion [6, 7, 14, 29–31] and may explain loss of beta cell function in response to continuous elevation of blood glucose in type 2 diabetes. In islets from diabetic mice or type 2 diabetes organ donors, mitochondrial energy metabolism is defective as demonstrated by reduced expression of subunits of respiratory chain complexes and a dramatic reduction in basal and glucose-induced respiration [32, 33]. In addition, mitochondria appear swollen with altered cristae and are unable to augment ATP in response to glucose [31, 34].

Glucose-induced impairment of beta cell function can be reproduced in vitro. After culture under high glucose conditions, the insulin content is reduced and islets display increased basal insulin secretion but only a poor or no response to maximal glucose stimulation [27, 29, 35, 36]. Here we studied early metabolic and mitochondrial adaptations to chronic glucose stress in human pancreatic beta cells.

## Methods

### Reagents, constructs and cell lines

Chemicals were from Sigma-Aldrich (Switzerland), Invitrogen (Switzerland), VWR, Tocris (Switzerland) or Thermo Fisher Scientific (USA). The YC3.6_cyto_ pcDNA3 construct was kindly provided by A. Miyawaki (Riken Brain Science Institute, Wako, Japan) and R. Tsien (University of California, San Diego). Adenoviruses were produced by Sirion Biotech (Germany) in CAP cells. Mycoplasma-free INS-1E cells were obtained from CBW (University of Geneva, Switzerland) and were cultured and analysed as described previously [14].

### Human islet culture and dissociation

Human islets from non-diabetic deceased donors were purchased from Tebu-bio (Le Perray-en-Yvelines, France). Donors had consented to donate organs for medical research. Only human islets from nondiabetic donors with a BMI between 20 and 30 were used (see ESM; checklist for reporting human islet preparations used in research). The use of human islets was approved by the local independent ethics committee of the Canton of Vaud (Switzerland). Islet clusters were cultured as described [37] in MEM medium containing 5.6 mmol/l glucose (no. 11095–080; GIBCO [USA]). The medium was supplemented with 5% (vol./vol.) heat-inactivated FBS (Chemie Brunschwig, Switzerland), GlutaMAX (no. 35050061; GIBCO [USA]), B-27 supplement (no. 17504044; GIBCO [USA]), 10 mmol/l HEPES and 1 mmol/l Na pyruvate (human islet medium). The antibiotics penicillin (50 μg/ml) and streptomycin 100 μg/ml were included. Plastic or glass surfaces were coated with 50 μl HBSS with Mg^2+^/Ca^2+^ (no. 14025–092; Life Technologies [USA]) containing 50 μg /ml collagen IV (no. C5533; Sigma-Aldrich [Switzerland]). Coated surfaces were washed with HBSS. Islets were dissociated in pre-warmed 0.05% trypsin/EDTA solution (500 μl) for 4 min. Human islet medium was added to stop digestion. Dissociated cells were centrifuged and re-suspended in human islet medium (50 μl/well) and seeded. Additional medium was added 24 h later.

### Human islet adenovirus infection

Attached islet clusters were infected 3–4 days after plating. Samples were infected with (20–40 infection units/cell) for 90 min in human islet medium at 37°C. Cells were washed with PBS (no. 10010–015; GIBCO [USA]) before adding fresh medium. The experiments were performed 24 h after infection.

### Transmission electron microscopy

Dissociated islet cells (50 islet equivalents per well) were seeded, allowed to attach on collagen IV-coated coverslips (no. 174969; Nalge Nunc [USA]) and cultured for 4 days. The cells were then washed twice briefly with PBS pH 7.4 and fixed for 1 h in PBS containing 2% paraformaldehyde and 2.5% glutamine at room temperature. The cells were washed three times at 4°C in cacodylate buffer (0.1 mol/l, pH 7.4). The samples were postfixed twice for 40 min at room temperature in cacodylate buffer (0.1 mol/l, pH 7.4) with 1% osmium tetroxide. The cells were washed for 5 min in distilled water and stained for 40 min in 1% uranyl acetate. The cells were then washed for an additional 5 min in distilled water and dehydrated in a graded alcohol series (1 × 50%, 1 × 70%, 2 × 96%, 2× 100%). The cells were embedded in Durcupan (Sigma-Aldrich [Switzerland]). The samples were placed on coated glass slides and left overnight at 65°C. Sections cut with a diamond knife (50 nm thick) were collected onto Pioloform support films on single-slot copper grids, contrasted with lead citrate and uranyl acetate. Images were taken with a transmission electron microscope at 80 kV (Tecnai Spirit [USA], FEI Company with Eagle CCD camera).

### Metabolite analysis

Human islets were dissociated and seeded in 12-well plates (TPP [Switzerland] no. 92012). Islet cells (1000 islet equivalents per well) were cultured in human islet medium for 4 days. To prevent the proliferation of fibroblasts, cytarabine (3 μmol/l) was added to the medium. The islet clusters were washed twice rapidly with 0.9% (wt/vol.) NaCl and were snap frozen in liquid nitrogen and stored at −80°C. For the extraction, a cold biphasic method was employed, as described elsewhere [38].

The dried extracts were dissolved in 20 μl 60% acetonitrile and injected (5 μl) into a Vanquish UHPLC + focused LC system (Thermo Scientific [USA]), equipped with a hydrophilic LC column (ZIC-pHILIC column, 100 × 2.1 mm, 5 μm, with a ZIC-pHILIC guard column 20 × 2.1 mm, 5 μm, both from Merck Sequant [Germany]). The separation was achieved by applying a linear solvent gradient in decreasing organic solvent (90–25%, 15.5 min) at 0.2 ml/min flow rate and 35°C. Aqueous 10 mmol/l ammonium acetate with 0.04% (vol./vol.) ammonium hydroxide, and acetonitrile, were used as mobile phases. The eluting metabolites were analysed on an Orbitrap Fusion Lumos mass spectrometer (Thermo Scientific [USA]) equipped with a heated electrospray ionisation (H-ESI) source. The *m*/*z* of the metabolites was assessed in the Orbitrap analyser with on-the-fly positive and negative ion mode switching using a resolution of 60,000 at *m*/*z* of 200. The spray voltages were 3500 V and 3000 V for positive and negative mode, respectively. The sheath gas was 20 arbitrary units (AU), and the auxiliary gas was kept 15 AU. The temperature of the vaporiser was 280°C and the temperature of the ion transfer tube was 310°C. Instrument control and peak integration was conducted with the Xcalibur 4.2.47 software (Thermo Scientific [USA]). Metabolites were identified according to their exact mass and the signal intensities were normalised to ^13^C internal standard and protein content.

### Static insulin secretion

Dissociated islets (20 islet equivalents/well) were plated on collagen IV-coated 24-well plates (no. 92024; TPP [Switzerland]) and cultured for 4 days in human islet medium. The cells were washed three times in Krebs–Ringer HEPES buffer (KRBH) containing 1 mmol/l glucose and 0.1% BSA (no. 268131000; Acros Organics [USA]) and maintained in this buffer for 1 h at 37°C. The cells were then washed once and incubated in KRBH containing 1 mmol/l glucose and 0.1% BSA for 30 min at 37°C (basal insulin secretion). The cells were then stimulated with 16.7 mmol/l glucose in KRBH containing 0.1% BSA for 30 min at 37°C (glucose-induced insulin secretion). Insulin content was determined after acid ethanol (1.5% HCl/70% ethanol) extraction overnight at 4°C. An ELISA kit (no. A0510596; SpiBio, France) was used to measure insulin.

### Single-cell imaging

Cells were imaged on a DMI6000 B inverted fluorescence microscope, using an HCX PL APO 40× / 1.40–1.30 NA oil immersion objective (Leica Microsystems, Germany) and an Evolve 512 back illuminated CCD with 16 × 16 pixels camera (Photometrics, AZ, USA).

Cytosolic Ca^2+^signals were recorded with the Cameleon sensor YC3.6 [39] expressed under the rat insulin promoter (Ad-RIP-YC3.6). Cells were excited at 435 nm. Ca^2+^ signals are measured by fluorescence resonance energy transfer as an increase in fluorescence emission at 535 nm and a decrease at 480 nm. The data is expressed as the ratio of 535/480 fluorescence emission. The relative changes of ratio are expressed compared to the ratio before glucose stimulation. Cytosolic ATP was measured using ATeam (Ad-CMV-cytoATeam) [15].

### Oxygen consumption measurements

Seahorse plates (96-well format; no. 101085–004; Agilent [USA]) were coated with collagen IV. Dissociated islet cells (50 islet equivalents per well) were seeded and maintained in human islet medium containing cytarabine (3 μmol/l; no. 16069; Cayman Chemical [USA]). For the experiment, the medium was changed to KRBH containing 1 mmol/l glucose and conditions were maintained for 30 min at 37°C. Oxygen consumption ($\dot{V}{\mathrm{O}}_2$) was measured in an XF96 instrument (Seahorse Biosciences, MA, USA) at 37°C. The $\dot{V}{\mathrm{O}}_2$ in permeabilised cells was measured as previously described [40].

### Statistics

The significance of differences between means was established using the Student’s *t* test for unpaired samples and two-tailed distribution. Analyses involving multiple donors were performed using linear mixed-effect models (LMMs) [41], to assess the differences in metabolic readouts between treated and control cells. Those models adjusted analyses for HbA_1c_ (as a fixed effect) and donor (as a random effect). LMMs are optimal to handle the dependencies between replicates/donors [42]. LMMs were fitted using the nlme R package [43] (version 3.1–145).

Joint adjustment for age, BMI and HbA_1c_ (as fixed effects) could not be performed with the same model due to singularity issues. However, independent models with adjustments for either BMI or age led to the same conclusions as the models only adjusting for HbA_1c_. Multivariate analyses of metabolomics were performed using sparse partial least-square discriminant analyses (sPLS-DA) [41]. Models were fitted on two components, allowing a maximum of ten metabolites per component. Data was scaled to zero-mean and unit variance. Stability of the models was tested using leave-one-out validation. Analyses were performed using the MixOmics R package [43] (version 6.10.9). Statistical analyses were performed using the R language [43] (version 3.6.1).

The samples were not randomised and the experimenters were not blinded to whether the human islet samples were cultured under control or elevated glucose conditions.

## Results

### Glucose stress lowers insulin secretion from human islet clusters

**Fig. 1:**
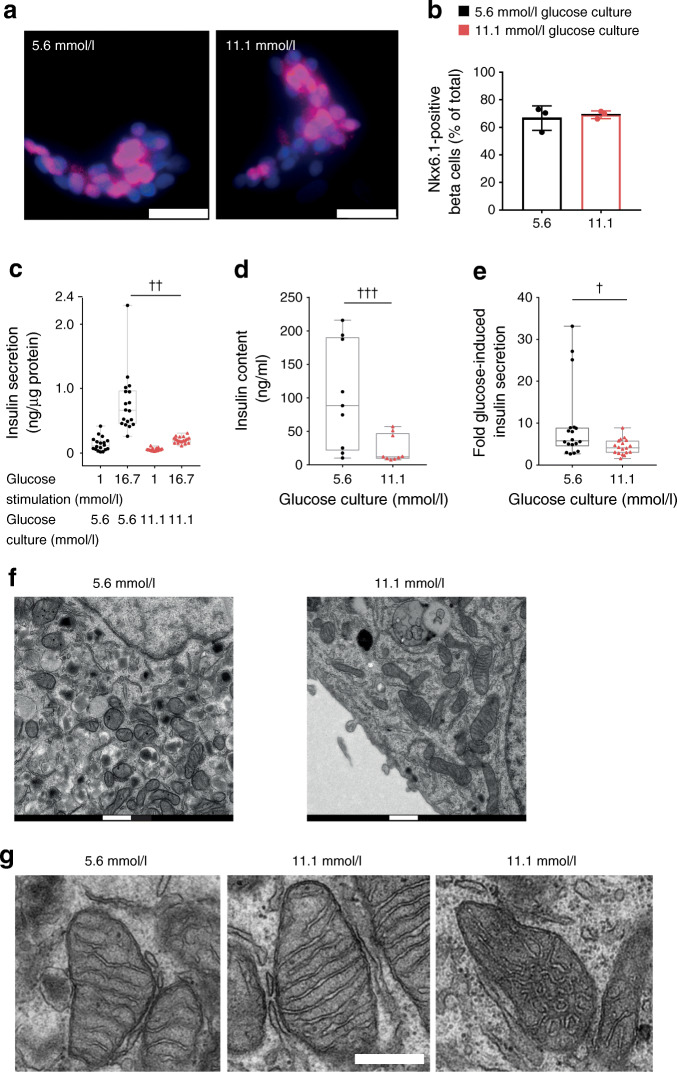
Modest beta cell dysfunction in response to glucose stress. (**a**) Nkx6.1 immunofluorescence labelling (pink) in human islet clusters grown for 4 days in culture medium supplemented with either 5.6 mmol/l or 11.1 mmol/l glucose. Nuclei were stained with DAPI (blue). Scale bar, 25 μm. (**b**) The percentage of Nkx6.1-positive cells was determined after 4 day culture in 5.6 mmol/l glucose (black bar) or 11.1 mmol/l glucose (red bar). The mean from three donors ± SD is shown; >25 images per donor and condition were quantified. Statistical significance was calculated using a *t* test (not significant). (**c**) Acute glucose-induced insulin secretion was measured from human islet clusters following 4 days of culture in islet medium containing either 5.6 mmol/l (black circles) or 11.1 mmol/l glucose (red triangles). Basal conditions were established in KRBH containing 1 mmol/l glucose. Insulin secretion was measured after 30 min in 1 mmol/l glucose followed by 30 min stimulation with 16.7 mmol/l glucose, as indicated. Results are from three donors (*n* = 18). LMM, ^††^*p* < 0.01. (**d**) Insulin content in human islet clusters was measured in separate samples from the same three donors as shown in (**c**) in KRBH containing 1 mmol/l glucose (*n* = 9). LMM, ^†††^*p* = 2.24 × 10^−8^. (**e**) Insulin secretion was expressed as fold change of glucose-induced (16.7 mmol/l) insulin secretion divided by basal (1 mmol/l glucose) secretion. Results are from three donors (*n* = 18). LMM, ^†^*p* = 0.02. (**f**) Electron micrograph of a beta cell in an islet cluster grown for 4 days in 5.6 mmol/l or 11.1 mmol/l glucose. (**g**) Cristae morphology of mitochondria in beta cells grown in 5.6 mmol/l glucose or 11.1 mmol/l glucose. Also shown is a representative image of a small subset of mitochondria from cells grown at 11.1 mmol/l glucose displaying perturbed cristae morphology. Scale bars, 500 nm

We looked for early molecular events leading to beta cell dysfunction that occur as a result of impaired glucose control in a recently developed cellular model of human islet clusters [37]. Cells were cultured for 4 days in either 5.6 mmol/l glucose (control condition) or 11.1 mmol/l glucose (moderate glucose stress). The fraction of cells expressing the beta cell transcription factor Nkx6.1 was preserved after elevated glucose culture but insulin secretion compared with control was strongly reduced (Fig. 1a–c). Culture of islet clusters in 11.1 mmol/l glucose medium markedly reduced the insulin content (Fig. 1d) and modestly lowered fold glucose-induced insulin secretion (Fig. 1e). Furthermore, given the strongly reduced insulin content, basal insulin secretion, when expressed as a fraction of the content, was significantly higher in glucose-stressed islet clusters (data not shown).

### Ultrastructural defect in a subset of beta cell mitochondria following glucose stress

After glucose stress, the number of insulin granules per beta cell area was strongly reduced in agreement with the reduced insulin content (Fig. 1d,f). Large/swollen round mitochondria with disorganised cristae have been described in islet beta cells from human donors with type 2 diabetes or after culture under glucotoxic conditions [31, 34]. We did not find such severe phenotypes after culture in 11.1 mmol/l glucose (Fig. 1f,g). Only in a small subset of beta cell mitochondria did we observe perturbed cristae morphology (Fig. 1g). This mitochondrial phenotype could be a first hint of mitochondrial decompensation due to chronic glucose stress.

### Chronic glucose stress enhances basal respiration and impairs glucose responsiveness of mitochondria

**Fig. 2:**
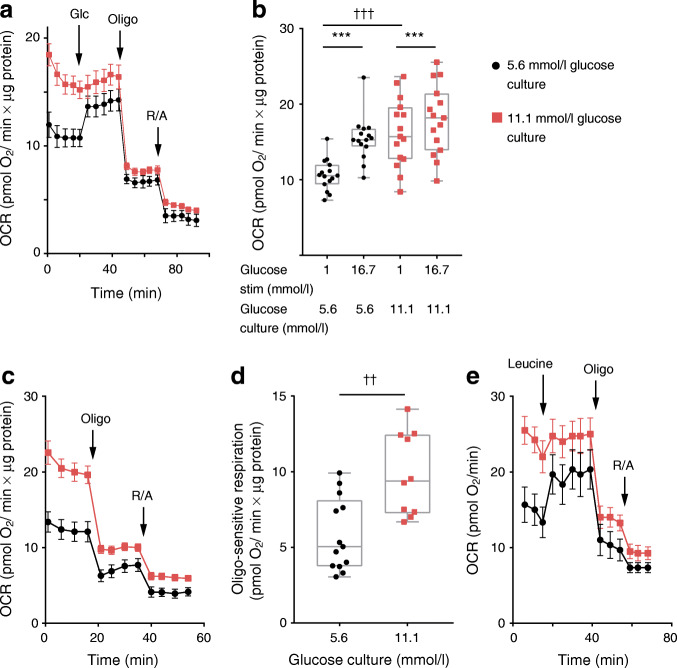
Elevated glucose culture enhances basal ATP-synthase-dependent respiration and blunts the glucose-induced respiratory response. Respiration rate was analysed in human islet clusters grown for 4 days in 5.6 mmol/l (black circles) or 11.1 mmol/l glucose culture (red squares). For respiratory analysis, resting conditions were established in KRBH containing 1 mmol/l glucose. After 45 min in KRBH containing 1 mmol/l glucose, beta cells were stimulated with glucose (Glc; 16.7 mmol/l; **a**, **b**) or leucine (10 mmol/l; **e**) as indicated by the arrows. Following the nutrient response, respiration was sequentially inhibited with oligomycin (Oligo; 50 μg/ml) and rotenone (50 μmol/l) plus antimycin A (50 μg/ml) (R/A). (**a**) Respiratory response of human islet clusters from a single donor. Respiration rates per total islet protein are shown (mean ± SEM; *n* = 5). Similar results were obtained with islet clusters from two other donors. (**b**) Quantification of the respiratory responses of human islet clusters from three donors (*n* = 15). Human islet clusters were grown and stimulated with glucose (16.7 mmol/l) as indicated. LMM, ^†††^*p* < 0.001, ***p < 0.001. (**c**) ATP-synthase (oligo)-dependent respiration under basal glucose (1 mmol/l) conditions. The mean ± SEM (*n* = 5) from a single donor is shown. Similar results were obtained with two additional donors. (**d**) ATP-synthase-dependent respiration in human islet clusters was calculated as the difference of the respiratory rate before and after addition of oligo (50 μg/ml). Results were obtained from human islet clusters from three donors (*n* = 13 for control and *n* = 10 for samples grown in 11.1 mmol/l glucose). LMM, ^††^*p* = 0.0012. (**e**) Leucine (10 mmol/l)-induced respiratory response of human islet clusters. Results are the mean ± SEM (*n* = 5) from a single donor. OCR, oxygen consumption rate; stim, stimulation

Acute glucose stimulation accelerates mitochondrial respiration in pancreatic beta cells [14, 44–46], shown here in control human islet clusters (Fig. 2a). In islet clusters cultured under chronically elevated glucose, basal respiratory rates per total protein were markedly elevated compared with the rate observed in islets cultured under control conditions (increased by 52.4%, *p* < 0.001) (Fig. 2a,b). To test whether augmented basal respiration is due to mitochondrial dysfunction, such as uncoupling, we measured ATP-synthase-dependent respiration under resting glucose conditions. Addition of the ATP synthase inhibitor oligomycin showed that, under basal glucose conditions, coupled respiration was markedly higher following glucose stress (Fig. 2c,d). Similar results were obtained in the rat insulinoma cell line INS-1E: chronic culture in media containing 11.1 or 16.7 mmol/l glucose caused a stepwise increase of basal respiration when compared with INS-1E cells grown in 5.6 mmol/l glucose (electronic supplementary material [ESM] Fig. 1). We conclude that glucose stress causes a gain of function at the level of mitochondrial energy metabolism.

While the absolute respiratory rates were consistently higher in islet clusters after glucose stress, the fold glucose-induced respiratory responses were small (Fig. 2a,b). After glucose stress, the mitochondria were also less sensitive to acute leucine (10 mmol/l) stimulation, demonstrating a general loss of mitochondrial nutrient sensitivity (Fig. 2e). In INS-1E cells, glucose responsiveness at the level of mitochondrial respiration was preserved (even slightly elevated) after culture in 11.1 or 16.7 mmol/l glucose (ESM Fig. 1). INS-1E cells appear protected from glucose-induced dysfunction.

**Fig. 3:**
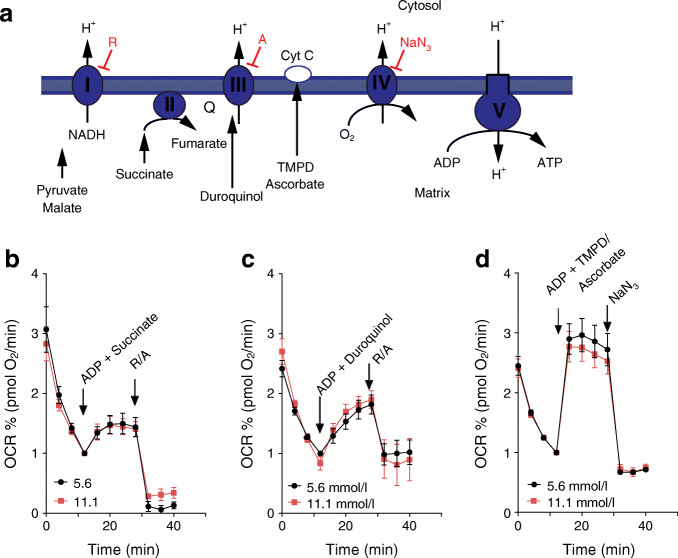
The activities of complex II, III and IV of the respiratory chain are normal in islet cells after culture in conditions of chronic elevated glucose. (**a**) Scheme of the respiratory chain and the substrates and inhibitors used to study respiration in permeabilised cells. (**b**–**d**) Respiration rates of permeabilised islet cluster cells after growth for 4 days in medium containing 5.6 mmol/l (black circles) or 11.1 mmol/l glucose (red squares). Complex II (**b**) (12 measurements from 3 donors), complex III (**c**) (10 measurements from 3 donors) and complex IV (**d**) (18 measurements from 4 donors) activity was measured after stimulation of respiration with ADP (10 mmol/l) in combination with the complex-specific substrates: succinate (10 mmol/l) for complex II; duroquinol (0.5 mmol/l) for complex III); or TMPD (0.5 mmol/l) plus ascorbate (2 mmol/l) for complex IV. Subsequently, the inhibitors rotenone (1 μmol/l) plus antimycin A (20 μmol/l) or NaN_3_ (20 mmol/l) were added to block complex activity. The mean ± SEM are shown. A, antimycin A; Cyt C, cytochrome c; OCR, oxygen consumption rate; Q, ubiquinol; R, rotenone; TMPD, tetramethylphenylendiamin

In permeabilised human islet cells, the activity of respiratory chain complexes was unchanged after glucose stress conditions (Fig. 3). Our data show that enhanced basal respiration in islet cells after chronically elevated glucose culture was not due to a general induction of respiratory chain activity or elevated mitochondrial mass.

### Poor glucose-induced ATP formation and an elevated basal ATP/ADP ratio in islet cells following glucose stress

**Fig. 4:**
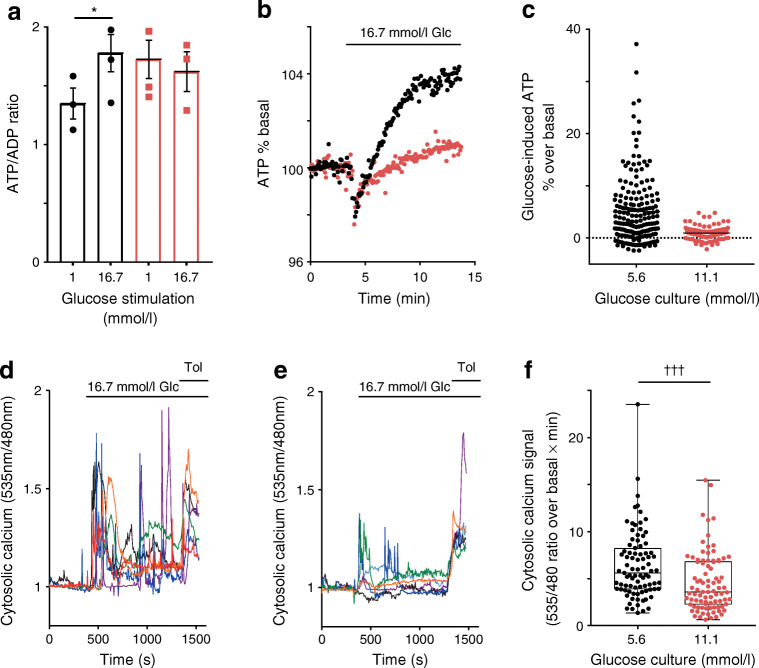
Elevated basal ATP/ADP ratio and blunted ATP and Ca^2+^ responses to glucose in islet cells after culture under conditions of chronic elevated glucose. (**a**) Extracts from islet clusters were analysed by LC–MS and the ATP/ADP ratios were calculated from ATP and ADP signals. Islet clusters were cultured in 5.6 mmol/l glucose (black bars) or 11.1 mmol/l glucose (red bars) and equilibrated in KRBH containing 1 mmol/l glucose, followed by 15 min incubation in the presence of either 1 mmol/l glucose or 16.7 mmol/l glucose, as indicated. The mean ATP/ADP ratio ± SEM in samples from three islet donors is shown. LMM, *p < 0.05. (**b**) Human islet clusters were infected with Ad-CMV-cytoAteam 1 day prior to the experiment. Cells were equilibrated in basal conditions (1 mmol/l glucose) and stimulated with glucose (Glc; 16.7 mmol/l), as indicated. Relative cytosolic ATP signals were monitored over time. Mean ATP responses to glucose of cells from three donors cultured in 5.6 mmol/l (black circles) or 11.1 mmol/l (red circles) are shown and expressed as percentage of the signal ratio under basal conditions. (**c**) Quantification of the Ateam responses in individual cells 10 min after initiation of glucose stimulation of islet clusters grown in control conditions (5.6 mmol/l glucose; 185 cells) or under chronic elevated glucose (11.1 mmol/l; 161 cells) conditions. (**d**–**f**) Human islet cells were infected with Ad-RIP-YC3.6 for beta cell-specific cytosolic Ca^2+^ measurements. After 35 min in KRBH containing 1 mmol/l glucose, the cells were stimulated with glucose (16.7 mmol/l) and, 15 min later, with tolbutamide (Tol; 100 μmol/l), as shown. Cytosolic Ca^2+^ rose after 5.6 mmol/l glucose culture (control) (**d**) and 11.1 mmol/l glucose culture (**e**). Different colours were used for the individual cells responding to glucose. (**f**) Quantification of cytosolic Ca^2+^ signals of individual cells from three donors corresponding to the conditions shown in (**d**) (5.6 mmol/l glucose; 86 cells) and (**e**) (11.1 mmol/l; 81 cells). The AUC was determined as the increase of the YC3.6 emission ratio (535 nm/480 nm) over basal during the first 3 min after glucose stimulation. Analysis of each cell is shown as a single data point. Mean plus minus quartiles are also indicated. LMM, ^†††^*p* < 0.001

After chronic glucose stress, the ATP/ADP ratio in islet cells remained elevated after returning the cells to 1 mmol/l glucose (Fig. 4a; 27.8% [*p* < 0.05] higher after chronic glucose culture compared with control), consistent with elevated basal ATP-synthase-dependent respiration (Fig. 2c,d). In control islet cells cultured in 5.6 mmol/l glucose, the ATP/ADP ratio was increased following stimulation with 16.7 mmol/l glucose (Fig. 4a). After glucose stress, islet clusters were unable to increase their ATP/ADP ratio in response to 16.7 mmol/l glucose (Fig. 4a). The poor ATP response to glucose was also observed in kinetic experiments using the ATP sensor ATeam. Compared with control islet cells, the glucose response was slow and of small amplitude (Fig. 4b,c). Over the time frame studied here, islet cells after chronic glucose culture are unable to return their ATP/ADP ratio to non-stimulatory levels.

### Defective glucose-dependent cytosolic Ca^2+^ signalling following glucose stress

After glucose stress, glucose-induced Ca^2+^ signalling was strongly reduced compared with control (Fig. 4d,e and ESM Fig. 2a). Under resting conditions, cytosolic Ca^2+^ transients were absent (Fig. 4d,e). Tolbutamide, which strongly depolarises the plasma membrane, was still able to induce Ca^2+^ signalling (ESM Fig. 2b). Impaired Ca^2+^ signalling is due to reduced nutrient-dependent activation of beta cells after chronic glucose stress.

### Glucose-derived metabolites were augmented following glucose stress

**Fig. 5:**
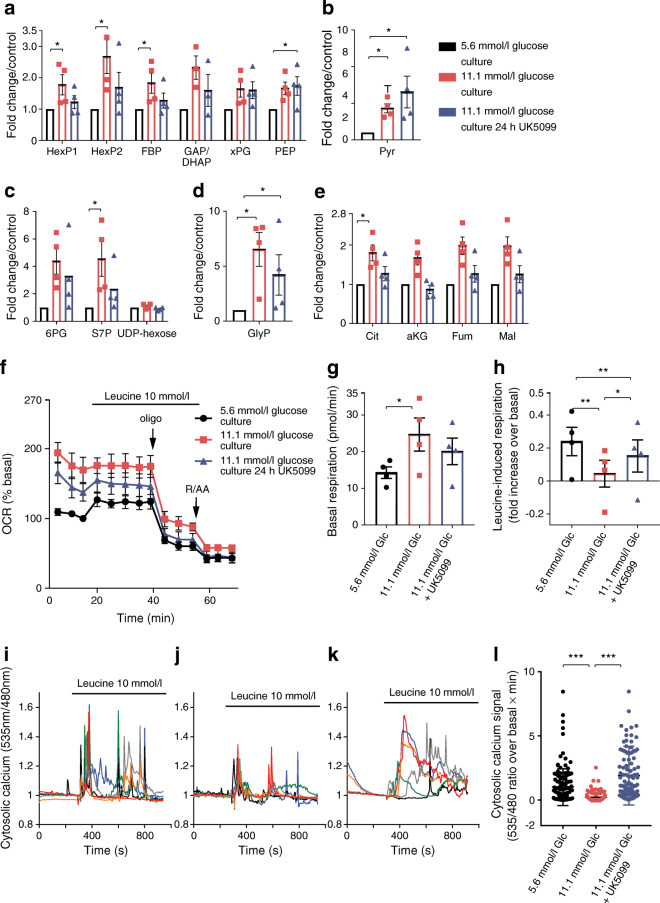
Inhibition of mitochondrial pyruvate transport partially reverses changes caused by chronic glucose stress. Islet clusters were cultured under either control conditions (5.6 mmol/l glucose; black bars and symbols) or chronic elevated glucose conditions (11.1 mmol/l glucose; red bars and symbols). In a second set of cells cultured in elevated glucose, mitochondrial pyruvate uptake was inhibited with UK5099 (10 μmol/l) for 24 h before the experiment (blue bars and symbols). (**a**–**e**) Metabolites were extracted after culture of islet clusters. Augmentation of intracellular metabolite levels were expressed as fold increase over the values in control culture conditions (5.6 mmol/l glucose). HexP1 includes glucose-6-phosphate, HexP2 includes glucose-1-phosphate and fructose-6-phosphate, and xPG includes 3-phosphoglycerate and 2-phosphoglycerate. FBP, fructose bis-phosphate, GAP, glyceraldehyde 3-phosphate; DHAP, dihydroxyacetone phosphate; PEP, phosphoenolpyruvate; Pyr, pyruvate; 6PG, 6-phosphogluconate; S7P, sedoheptulose 7-phosphate; UDP, uracil-diphosphate; GlyP: glycerol-3-phosphate; Cit, citrate; aKG, alpha-ketoglutarate; Fum, fumarate; Mal, malate. The mean from four donors ± SEM is shown. (**f**) Leucine (10 mmol/l)-induced respiration in islet clusters cultured as described above. Islet cells were incubated for 30 min in KRBH containing 1 mmol/l glucose before initiating the experiment. (**g**, **h**) Quantification of basal (**g**) and leucine-induced respiration (**h**) as shown in (**f**). Results are the mean from four donors ± SEM. (**i**–**k**) Leucine-induced Ca^2+^ signalling in islet beta cells following culture under control conditions (**i**), chronic elevated glucose conditions (**j**) or conditions of chronic elevated glucose with the addition of UK5099 (10 μmol/l) to the culture medium for the last 24 h (**k**). For the UK5099-treated cells, the same inhibitor concentration was maintained during the experiments. Data representation and analysis was as described for Fig. 4d–f. (**l**) Quantification of cytosolic calcium responses in individual beta cells as AUC from experiments shown in (**i**−**k**). For each condition, at least 120 cells from three islet donors were analysed. *p* values were obtained by Student’s *t* test: *p < 0.05, **p < 0.01, ***p < 0.001. Glc, glucose; Oligo, oligomycin

Extraction of intracellular metabolites after glucose stress revealed that several intermediates of the glycolytic pathway (Fig. 5a,b), the pentose phosphate pathway (PPP) (Fig. 5c) and the TCA cycle (Fig. 5e) were elevated significantly (or were increased to a non-statistically significant extent) as compared with control islet cultures (see ESM Fig. 3 for metabolites analysed). The most strongly augmented metabolite following high glucose culture was glycerol 3-phosphate (Fig. 5d). Chronic glucose stress led to a global adaptation of central metabolism.

### Inhibition of mitochondrial pyruvate transport normalises metabolite changes and beta cell dysfunction caused by chronic elevated glucose

To protect beta cell mitochondria from potential substrate oversupply during chronic elevated glucose culture, we used the mitochondrial pyruvate transporter inhibitor UK5099 [47]. In acute experiments, UK5099 causes maximal inhibition of insulin secretion at 150–250 μmol/l [12]. Chronic treatment for 24 h with UK5099 (100 μmol/l) had toxic effects on human islet cells (data not shown). Here, we tested the effect of 10 μmol/l UK5099, which likely inhibits but does not completely block mitochondrial pyruvate uptake in human islets. UK5099 was included in the 11.1 mmol/l glucose medium for the last 24 h before the experiment. This treatment partially reversed several of the metabolite changes caused by glucose stress (Fig. 5a–e). Inhibition of mitochondrial pyruvate transport lowered the levels of the TCA cycle intermediates (Fig. 5e). In addition, several glycolytic intermediates (Fig. 5a), the two PPP metabolites 6-phosphogluconate and sedoheptulose 7-phosphate (Fig. 5c), and glycerol 3-phosphate (Fig. 5d), were reduced when UK5099 was included in the medium. Likely as a direct effect of mitochondrial pyruvate transport inhibition pyruvate was slightly increased by addition of UK5099 (Fig. 5b). This analysis suggested that UK5099 (10 μmol/l) lowers those metabolites elevated by chronic high glucose culture (except for pyruvate) but this correction did not reach significance when studying metabolites individually. We therefore performed an sPLS-DA to compare samples based on the overall changes in metabolite concentrations (ESM Fig. 4). The three groups (5.6 mmol/l glucose, 11.1 mmol/l glucose, and 11.1 mmol/l glucose plus 10 μmol/l UK5099) could be distinguished. sPLS-DA was also able to discriminate between samples grown under chronic glucose stress with or without UK5099. This is likely due to the correction of several metabolite concentrations by UK5099 to approach the concentrations measured in the human islets cultured under control conditions. Good examples are α-ketoglutarate and citrate, the two metabolites that contributed most strongly to the separation of the two groups on the first dimension of the sPLS-DA (explaining already 30% of the variance), but several other metabolites make additional individual contributions (ESM Fig. 4).

Inclusion of UK5099 (10 μmol/l) during chronic glucose stress also partially restored the respiratory phenotype in human islets. After culture, the islet cells were allowed to equilibrate in 1 mmol/l glucose with or without continued presence of UK5099. Leucine was used to stimulate respiration, to determine mitochondrial nutrient responsiveness bypassing mitochondrial pyruvate uptake. After elevated glucose culture, the respiratory response to leucine (10 mmol/l) was blunted (Fig. 5f–h; see also Fig. 2e) and was partially restored when mitochondrial pyruvate transport was inhibited during culture (Fig. 5f,h). On the other hand, basal respiration was not corrected by 24 h of treatment with UK5099 (Fig. 5f,g) showing that mitochondria remained significantly overactivated.

UK5099 also improved leucine-induced cytosolic Ca^2+^ signalling (Fig. 5i–l). Leucine-induced Ca^2+^ signalling in cells cultured under control conditions (Fig. 5i) was strongly reduced in cells previously cultured in elevated glucose conditions (Fig. 5j) but was restored when the pyruvate transport inhibitor was added during the last 24 h of glucose stress (Fig. 5k,l).

## Discussion

It has been proposed that chronic overstimulation of the beta cell precedes beta cell failure along the trajectory of type 2 diabetes progression. Nutrient oversupply results in insulin secretion beyond the capacity of beta cells to maintain their normal insulin content [48]. Exposure of human beta cells to diabetic glucose conditions for several days lowers insulin content and the ability to release insulin from human islets both in vitro and in vivo [26, 35]. Similarly, impaired glucose handling may overstimulate the beta cell although the transition to beta cell failure may take years rather than just a few days. Here, we describe how islet cells adapt their glucose metabolism and mitochondrial function when exposed to 11.1 mmol/l glucose for several days. The observed reduction in insulin content and diminished glucose-induced insulin secretion are consistent with findings from earlier studies [27, 29, 35, 36, 49].

Islets exposed chronically to elevated glucose do not fail to respond to glucose but are more sensitive to glucose, already releasing insulin at basal glucose concentrations [35, 49, 50]. Loss of insulin content and a reduction in glucose-induced insulin secretion as observed here suggest that the islet beta cells are at an early stage along the trajectory of dysfunction.

After culture in 11.1 mmol/l glucose, we did not observe large/swollen round mitochondria with disorganised cristae, such as described in islet beta cells from human donors with type 2 diabetes or in rat islets cultured under severely glucotoxic conditions [31, 34]. The main rather surprising phenotype of mitochondria was a pronounced increase in basal respiration after glucose stress in both human islet clusters and INS-1E cells. This is the opposite of islets from diabetic donors, in which mitochondrial respiration and energy metabolism are strongly reduced [31–33]. The mechanisms of how glucose stress overactivates mitochondria and prevents them from returning to the resting state should be further studied as this phenomenon may represent an early maladaptive response leading to beta cell dysfunction.

Glucose-induced acceleration of respiration was impaired in human islet cells but preserved in rat INS-1E cells cultured under chronic glucose stress conditions. These conflicting results may be due to species differences. Human islets are more sensitive than rodent islets to glucotoxicity. For example, transplantation of human islets into immunocompromised mice revealed that under conditions of chronic hyperglycaemia or acute hyperglycaemia with insulin resistance human islet function is strongly impaired [51]. Mouse islet function in response to acute hyperglycaemia with insulin resistance was mostly preserved.

During chronic glucose overload, the beta cell diverts some excess carbons into other metabolic pathways in order to preserve glucose flux for the stimulation of insulin secretion. Such fuel detoxification pathways include the synthesis of fatty acids, cholesterol and cholesteryl esters, which can be exported from the beta cell [52]. In addition, harmful lipid intermediates formed contribute to glucotoxicity. Human and rodent beta cells may differ in the metabolic pathways dealing with fuel detoxification. In rat islets, about one-third of glucose-derived carbons give rise to glycerol, which can be released from the beta cell as part of the glucose detoxification mechanism mostly formed from glycerol 3-phosphate [52, 53]. Interestingly, we found in human islet cells that glycerol 3-phosphate is the metabolite most strongly induced after chronic glucose stress. Human beta cells express glycerol 3-phosphatase but, unlike rat beta cells, do not seem to express aquaporin-7, the transporter required for glycerol export [54, 55]. This detoxification pathway may, therefore, be less efficient in human beta cells, rendering them more glucose sensitive.

An additional mechanism of fuel detoxification in INS-1E cells is their ability to form and secrete lactate when challenged with glucose. This is not a mechanism of fuel detoxification in primary beta cells, which express extremely low amounts of lactate dehydrogenase and do not express monocarboxylate transporter-1 for lactate export [56, 57]. Such metabolic differences may explain the differential sensitivity of human beta cells and rat INS-1E cells to chronic glucose overload.

Mitochondria from islet cells cultured in elevated glucose are highly functional and maintain accelerated ATP-synthase-dependent respiration and an elevated ATP/ADP ratio at glucose concentrations below the physiological range. The elevated ATP/ADP ratio is insensitive to acute glucose stimulation. The inability of mitochondria to return to a resting state with maintained high ATP levels may explain the reported left shift of glucose-dependent insulin secretion after growth under glucose stress conditions [49, 50, 58]. The augmentation of the ATP/ADP ratio when glucose is low prevents beta cells from returning to a resting state in preparation for future glucose stimulation.

Several of the mitochondrial adaptations and alterations of stimulus–secretion coupling in beta cells can be explained by the changes in metabolites that occur after culture under chronic elevated glucose conditions. Interestingly, all measured TCA cycle intermediates were elevated during glucose stress (Fig. 5e), suggesting that anaplerosis is stimulated. The elevated levels of 6-phosphogluconate and sedoheptulose 7-phosphate further suggest that high glucose culture promotes metabolism along the PPP (Fig. 4c). Recent evidence shows that the PPP is indeed important for metabolism secretion coupling in part because it is an important source of NADPH a likely regulator of the amplification pathway of insulin secretion [21, 25, 59].

Accumulated glycerol 3-phosphate may be a side-product of accelerated glycolysis and/or the consequence of reduced consumption by potential downstream metabolic reactions. Elevated glycerol 3-phosphate may promote glycerol phosphate shuttle activity and serve as a substrate for acylglycerol biosynthesis, which plays a role in the control of insulin secretion [58, 60, 61].

We propose that beta cell mitochondria fail to cope with nutrient oversupply during glucose stress. Consequently, metabolism is partially redirected towards pathways branching away from glycolysis such as the PPP and the glycerol phosphate shuttle. To protect mitochondria from substrate overload, we have used the mitochondrial pyruvate transport inhibitor UK5099. Mitochondrial pyruvate uptake is essential for acute glucose-stimulated insulin secretion [11, 12]. Inhibition of mitochondrial pyruvate transport in islets exposed to chronic elevated glucose concentrations will inhibit insulin secretion but allow beta cells to recover from continuous overstimulation. We selected a UK5099 concentration (10 μmol/l) much below the concentration previously observed to cause maximal inhibition of insulin secretion (100–250 μmol/l) [12]. Chronic (24 h) addition of UK5099 (100 μmol/l) caused morphological changes and cell loss suggesting toxic effects. Chronic treatment with 10 μmol/l UK5099 seemed beneficial as it reversed several (but not all) changes induced by high glucose culture. Using sPLS-DA analysis, we found that the inhibition of mitochondrial pyruvate transport partly corrects the pattern of metabolite changes induced by chronic 11.1 mmol/l glucose culture.

Inhibition of mitochondrial pyruvate transport did not normalise basal respiration but partially restored nutrient responsiveness (leucine-induced Ca^2+^ signalling and respiration). Based on these findings, we propose that protecting mitochondria from nutrient overload should be pursued as a strategy to allow beta cells to recover from glucose stress.

Here, we show that metabolite changes and enhanced energy metabolism under resting glucose conditions are among the first signs of beta cell adaptation to chronic elevated glucose. We propose that chronically altered intracellular metabolites result in mitochondrial adaptations that prevent mitochondria from returning to a resting state. Glycerol 3-phosphate, pyruvate and other metabolites elevated in chronically activated beta cells may act as intracellular buffers preventing glucose responsiveness over the normal physiological range. Interfering with the chronic oversupply of nutrients to mitochondria may be a way to normalise glucose metabolism and prevent insulin hypersecretion followed by loss of insulin content and beta cell function. Such intervention may prevent the conversion from impaired fasting glucose and/or glucose tolerance to overt type 2 diabetes.

## Electronic supplementary material

ESM (PDF 5676 kb)

## Data Availability

The datasets generated during and/or analysed during the current study are available from the corresponding author on reasonable request.

## Abbreviations

AU: Arbitrary units
KRBH: Krebs–Ringer bicarbonate HEPES buffer
LMM: Linear mixed-effect model
PPP: Pentose phosphate pathway
sPLS-DA: Sparse partial least-square discrimination analysis
TCA: Tricarboxylic acid
