# Supplementary material for: Augmented mitochondrial energy metabolism is an early response to chronic glucose stress in human pancreatic beta cells
Source: Diabetologia. 2020 Sep 22;63(12):2628–40. doi: 10.1007/s00125-020-05275-5 (PMC7641954; doi:10.1007/s00125-020-05275-5)
Supplement: Supplementary file 1 — (PDF 5676 kb) [file 125_2020_5275_MOESM1_ESM.pdf]

ESM Fig. 1

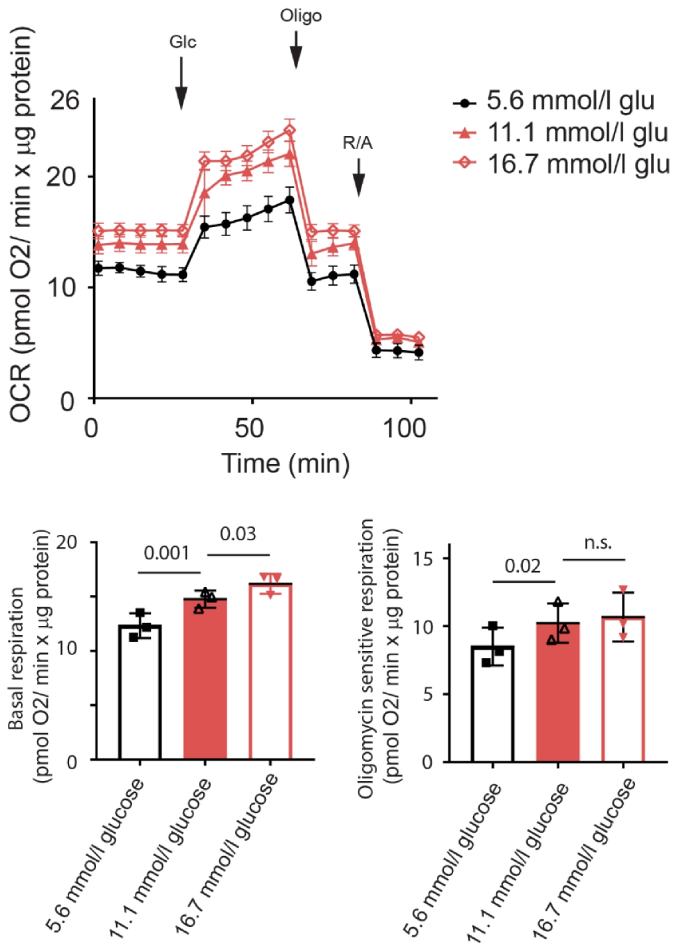

ESM Fig. 1. Elevated glucose culture augments basal respiration in INS-1E cells. INS-1E cells were grown for 3 days in RPMI culture medium containing 5.6 mmol/l glucose (black circles; black bars); 11.1 mmol/l glucose (red triangles; red bars) or 16.7 mmol/l glucose (red empty diamonds; red empty bar). The cells were changed to KRBH 2.5 mmol/l glucose. After an equilibration phase the cell were stimulated with 16.7 mmol/l glucose (Glc). Oligomycin (Oligo) and rotenone plus antimycin A (R/A) were added as described in the legend to Fig. 2. Basal respiration lower left panel and ATP-synthase dependent (oligomycin-dependent) respiration (lower right panel) were quantified. Shown are the average +/-SD from 3 experiments each performed in quadruplicate. p-values are given at the top of the bar graphs. n.s.: not significant.

ESM Fig. 2

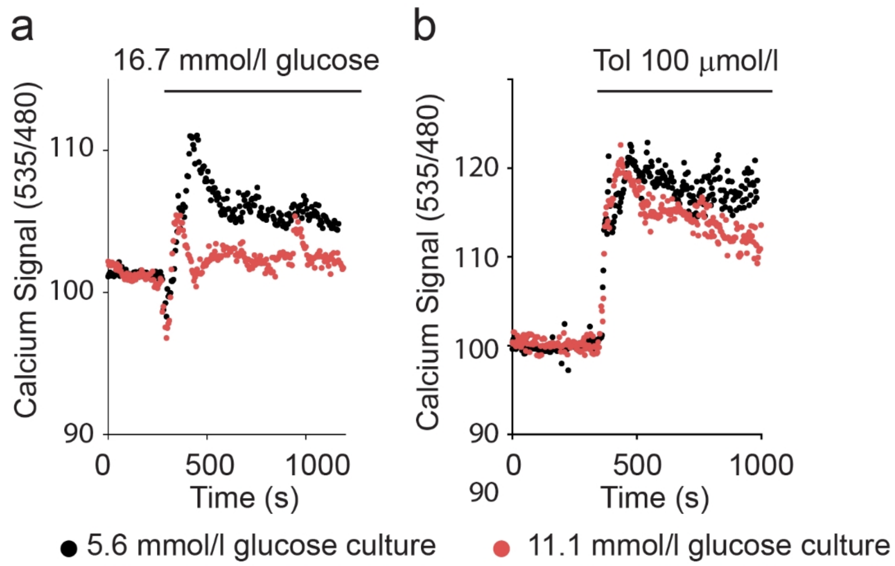

ESM Fig. 2. Effect of elevated glucose culture on Ca^{2+} signaling. Average cytosolic Ca^{2+} response to glucose (16.7 mmol/l; a) and tolbutamide (Tol; 100 μM; b) in beta cells after culture of human islet clusters for 4 days in 5.6 mmol/l (black trace) or 11.1 mmol/l glucose (red trace). Glucose responses were from > 80 cells from two islet donors for each condition. Tolbutamide responses were from > 35 cells from two islet donors for each condition.

ESM Fig. 3

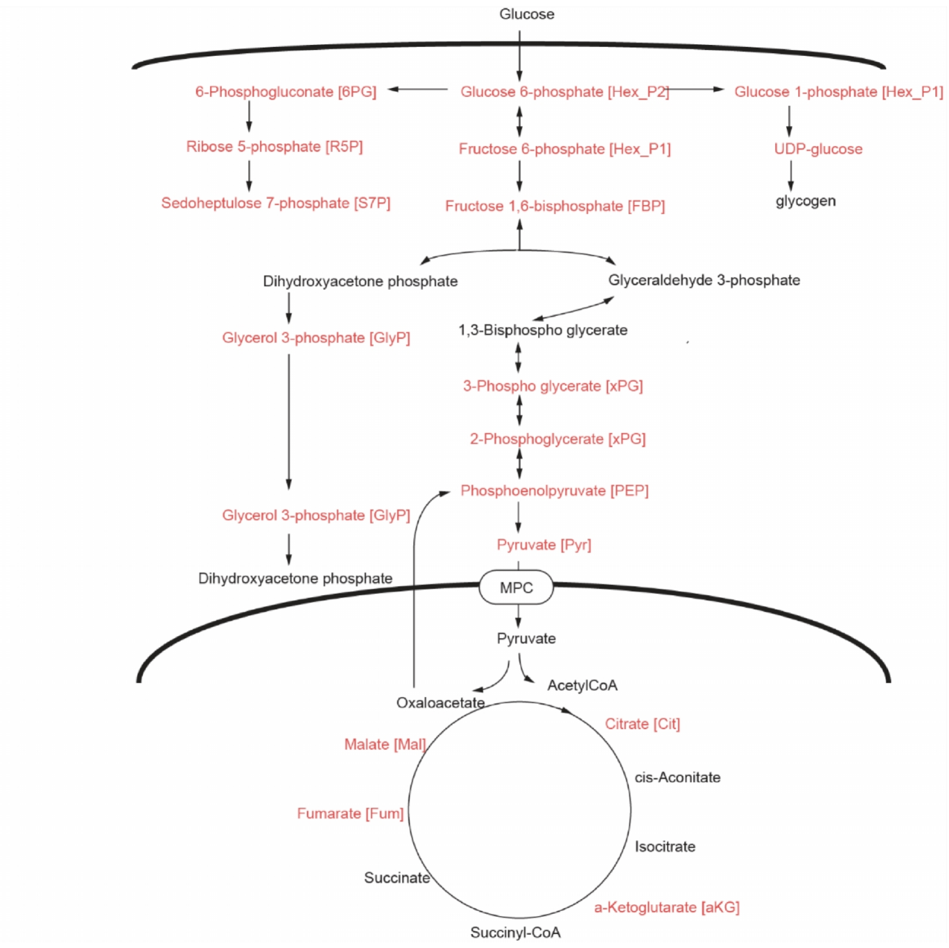

ESM Fig. 3. Metabolic pathways analyzed. Metabolites covered in this study are highlighted in red. Metabolites shown in black could not be consistently measured.

ESM Fig. 4

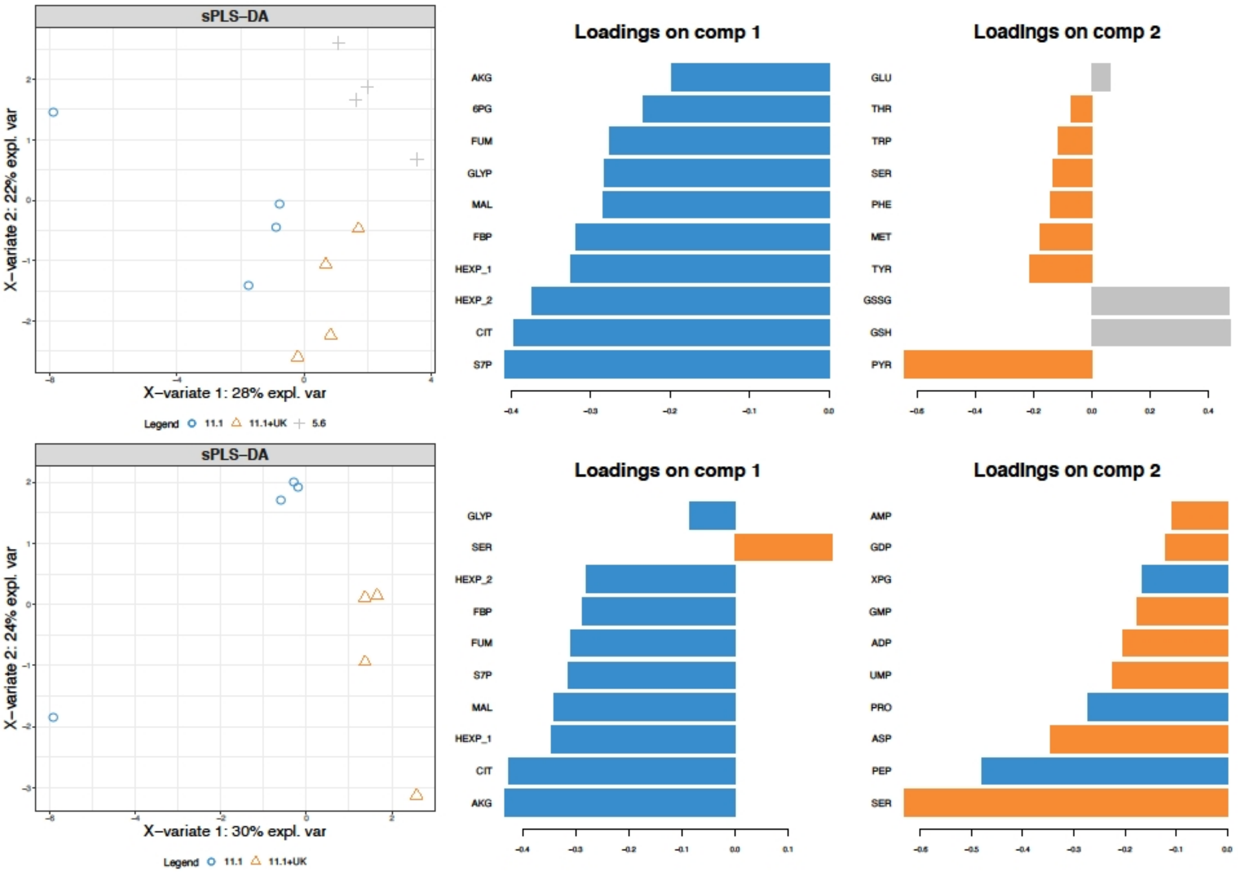

ESM Fig. 4. Metabolite changes induced by chronic 11.1 mmol/l glucose culture with and without inhibition of mitochondrial pyruvate transport. Sparse partial least square discriminant analysis (sPLS-DA) of metabolite in human islet clusters grown in 5.6 mmol/l glucose (grey crosses), 11.1 mmol/l glucose (blue circles) or 11.1 mmol/l glucose plus UK5099 (10µmol/l; orange triangles) as indicated in the upper left panel. The bottom left panel is from an analysis discriminating human islet cells cultured in 11.1 mmol/l glucose with (orange triangles) and without (blue circles) UK5099 (10 µmol/l). The plots on the left show the sPLS-DA projection on the two first components. The explained variance is indicated in the axis label. The barplots (middle and right panels) show the contribution (loadings) of identified metabolites on the 2 components. Metabolites are ranked by order of contribution on a component, and the bar color indicates whether an increase in this metabolite increases the probability to be within a specific treatment group. E.g. blue indicates that an increase in the metabolite levels increase the probability that the sample belong to the 11.1 mmol/l glucose stimulation group.

Human islet checklist 1

Diabetologia

Checklist for reporting human islet preparations used in research

Adapted from Hart NJ, Powers AC (2018) Progress, challenges, and suggestions for using human islets to understand islet biology and human diabetes. Diabetologia https://doi.org/10.1007/s00125-018-4772-2

Islet preparation	1	2	3	4	5	6	7	8[a]
**MANDATORY INFORMATION**								
Unique identifier	HP-17004-01	HP-17152-01	HP-18032-01	HP-18054-01	HP-16252-01	HP-16315-01	HP-17040-01	HP-17055-01
Donor age (years)	60	23	45	40	23	49	46	41
Donor sex (M/F)	M	M	M	M	M	M	M	M
Donor BMI (kg/m^2)	26	25.1	29.6	25	24.8	26.2	27.6	28
Donor HbA_{1c} or other measure of blood glucose control	5.9	5.6	5.1	5.3	5.3	5.2	5.4	5.3
Origin/source of islets[b]	Tebu-bio	Tebu-bio	Tebu-bio	Tebu-bio	Tebu-bio	Tebu-bio	Tebu-bio	Tebu-bio
Islet isolation centre	Prodo lab	Prodo lab	Prodo lab	Prodo lab	Prodo lab	Prodo lab	Prodo lab	Prodo lab
Donor history of diabetes? Please select yes/no from drop down list	No	No	No	No	No	No	No	No
**If Yes, complete the next two lines if this information is available**								
Diabetes duration (years)								
Glucose-lowering therapy at time of death[c]								
**RECOMMENDED INFORMATION**								
Donor cause of death	stroke	head trauma, MVA	chocking	MVA	anoxic event with CPR	head trauma, MVA	head trauma	head trauma
Warm ischaemia time (h)	0	0	14	0	27	0	0	0
Cold ischaemia time (h)	12	6	12.5	6.75	7	13	7.25	5.75
Estimated purity (%)								
Estimated viability (%)								
Total culture time (h)[d]								
Glucose-stimulated insulin secretion or other functional measurement[e]	Cytosolic calcium	Cytosolic calcium / Respiration (Seahorse)	Respiration (Seahorse)	Respiration (Seahorse) / metabolites	Insulin secretion	Insulin secretion	Cytosolic calcium / Insulin secretion	Insulin secretion
Handpicked to purity? Please select yes/no from drop down list	Yes	Yes	Yes	Yes	Yes	Yes	Yes	Yes
Additional notes	Electron microscopy (TEM)	Electron microscopy (TEM)	Immunofluore scence	Immunofluore scence				

[a]If you have used more than eight islet preparations, please complete additional forms as necessary
[b]For example, IIDP, ECIT, Alberta IsletCore
[c]Please specify the therapy/therapies
[d]Time of islet culture at the isolation centre, during shipment and at the receiving laboratory
[e]Please specify the test and the results

Human islet checklist 2

Diabetologia

Checklist for reporting human islet preparations used in research

Adapted from Hart NJ, Powers AC (2018) Progress, challenges, and suggestions for using human islets to understand islet biology and human diabetes. Diabetologia https://doi.org/10.1007/s00125-018-4772-2

Islet preparation	1	2	3	4	5	6	7	8[a]
**MANDATORY INFORMATION**								
Unique identifier	HP-17105-01	HP-17069-01	HP-17328-01	HP-18164-01	HP-18188-01	HP-18341-01	HP-19073-01	HP-17321-01
Donor age (years)	29	38	40	54	35	48	50	25
Donor sex (M/F)	M	M	F	M	F	M	M	M
Donor BMI (kg/m^2)	26.7	23.2	25	22.3	24	26.3	21.2	25.6
Donor HbA_{1c} or other measure of blood glucose control	5.4	5.6	5.7	5.8	4.8	5.1	5.5	5.7
Origin/source of islets[b]	Tebu-bio	Tebu-bio	Tebu-bio	Tebu-bio	Tebu-bio	Tebu-bio	Tebu-bio	Tebu-bio
Islet isolation centre	Prodo lab	Prodo lab	Prodo lab	Prodo lab	Prodo lab	Prodo lab	Prodo lab	Prodo lab
Donor history of diabetes? Please select yes/no from drop down list	No	No	No	No	No	No	No	No
**If Yes, complete the next two lines if this information is available**								
Diabetes duration (years)								
Glucose-lowering therapy at time of death[c]								
**RECOMMENDED INFORMATION**								
Donor cause of death	anoxic event	stroke	stroke	stroke	anoxic event with CPR	stroke	head trauma	MVA
Warm ischaemia time (h)	0.75	N. D.	0	0	20	N.D.	N.D.	0
Cold ischaemia time (h)	9	9	7.5	12.5	11.25	10.5	6.75	9.5
Estimated purity (%)								
Estimated viability (%)								
Total culture time (h)[d]								
Glucose-stimulated insulin secretion or other functional measurement[e]	Respiration (Seahorse) / Insulin secretion	Respiration (Seahorse)	Respiration (Seahorse) / Permeabilized Respiration /ATP	Respiration (Seahorse) / Cytosolic calcium	Respiration (Seahorse) / Cytosolic calcium / Metabolites	Respiration (Seahorse) /	Respiration (Seahorse) / Metabolites	ATP / Permeabilized respiration
Handpicked to purity? Please select yes/no from drop down list	Yes	Yes	Yes	Yes	Yes	Yes	Yes	Yes
Additional notes								

[a]If you have used more than eight islet preparations, please complete additional forms as necessary
[b]For example, IIDP, ECIT, Alberta IsletCore
[c]Please specify the therapy/therapies
[d]Time of islet culture at the isolation centre, during shipment and at the receiving laboratory
[e]Please specify the test and the results

Human islet checklist 3

Diabetologia

Checklist for reporting human islet preparations used in research

Adapted from Hart NJ, Powers AC (2018) Progress, challenges, and suggestions for using human islets to understand islet biology and human diabetes. Diabetologia https://doi.org/10.1007/s00125-018-4772-2

Islet preparation	1	2	3	4	5	6	7	8[a]
**MANDATORY INFORMATION**								
Unique identifier	HP-18017-01	HP-17036-01	HP-18094-01	HP-18095-01	HP-18132-01	HP-19003-01	HP-19038-01	HP-17307-01
Donor age (years)	57	59	39	29	45	63	55	55
Donor sex (M/F)	F	M	M	M	M	F	M	M
Donor BMI (kg/m^2)	21.4	26.9	NA	22.8	25	27	28.1	27.1
Donor HbA_{1c} or other measure of blood glucose control	5.8	5.8	5.4	5.5	5.1	5.8	5.0	5.8
Origin/source of islets[b]	Tebu-bio	Tebu-bio	Tebu-bio	Tebu-bio	Tebu-bio	Tebu-bio	Tebu-bio	Tebu-bio
Islet isolation centre	Prodo lab	Prodo lab	Prodo lab	Prodo lab	Prodo lab	Prodo lab	Prodo lab	Prodo lab
Donor history of diabetes? Please select yes/no from drop down list	No	No	No	No	No	No	No	No
**If Yes, complete the next two lines if this information is available**								
Diabetes duration (years)								
Glucose-lowering therapy at time of death[c]								
**RECOMMENDED INFORMATION**								
Donor cause of death	stroke	anoxic event with CPR	stroke	head trauma, fall	anoxic event	stroke	stroke	head trauma, fall
Warm ischaemia time (h)	0	0.75	0	0	N.D.	0	0	0
Cold ischaemia time (h)	6.5	16.5	8.2	9.25	10.2	8.8	8.5	8
Estimated purity (%)								
Estimated viability (%)								
Total culture time (h)[d]								
Glucose-stimulated insulin secretion or other functional measurement[e]	Permeabilized respiration / ATP	Cytosolic calcium	Metabolites	Metabolites	Metabolites	Metabolites	Cytosolic calcium / Metabolites	Permeabilized respiration
Handpicked to purity? Please select yes/no from drop down list	Yes	Yes	Yes	Yes	Yes	Yes	Yes	Yes
Additional notes								

[a]If you have used more than eight islet preparations, please complete additional forms as necessary
[b]For example, IIDP, ECIT, Alberta IsletCore
[c]Please specify the therapy/therapies
[d]Time of islet culture at the isolation centre, during shipment and at the receiving laboratory
[e]Please specify the test and the results
